# Around the Hospitals

**Published:** 1896-01-25

**Authors:** 


### AROUND THE HOSPITALS.

[Notice to the Managers of Institutions.—Special space is now reserved for the insertion of notices of hospital meetings and festivals. The Editor requests that all notices may reach The Hospital Office, 428, Strand, at least one week before the date of each meeting.]

**Derbyshire Royal Infirmary.**—The report of this infirmary just issued is the first since the institution has moved to its new quarters. We are glad to learn that progress and improvement are well maintained, not only in the structural condition but in the whole of the work. A larger number of patients have of course been able to receive benefit than was formerly possible. In the out-patients' department this is especially noticeable. Last year these amounted to 5,644, whilst in 1894 the number was only 4,374. Besides this an increase in casualty cases is noticeable, and a still larger one in dental cases. In-patients numbered 1,307, an increase of 262 in the same period. Public interest has been aroused to meet the expenses the larger number of patients entails. The funds show an increase of £887 irrespective of legacies. This and, indeed, yet more generous support will be necessary to meet the needs of the infirmary, and we doubt not that the efforts of the excellent secretary, Mr. Carnt, will be rewarded by a continuously increasing subscription list. The chief feature of the past year at the infirmary was the bazaar opened by the Duchess of Teck in aid of the furnishing fund. The results of the cheme amounted to £4,000, thus providing the funds needed. Several alterations in the bye-laws and regulations have been made, and a re-adjustment of hours in the out-patients' department has been found an improvement. The medical library has received a valuable present of 200 volumes from Dr. Ogle, the honorary consulting physician to the infirmary, and many other gifts have been received by the hospital from kind friends. The Convalescent Home at Holbrook has greatly aided the work of the hospital, and this is a direction in which aid is especially needed. The infirmary authorities are always glad to receive convalescent letters for other homes for the use of patients also. The income for the past year amounted to £5,218, and the expenditure to £6,677. The cost of in-patients per head is reckoned at £4 0s. 3½d. We regret to note that the Royal Derbyshire Infirmary, so much to the fore in most directions of hospital progress, has not so far adopted the uniform system of accounts, thus placing itself behind the times, and rendering stimulating comparison with other hospitals, on uniform lines, quite unsatisfactory.

